# A B Cell-Driven Autoimmune Pathway Leading to Pathological Hallmarks of Progressive Multiple Sclerosis in the Marmoset Experimental Autoimmune Encephalomyelitis Model

**DOI:** 10.3389/fimmu.2017.00804

**Published:** 2017-07-11

**Authors:** Bert A. ’t Hart, Jordon Dunham, Bart W. Faber, Jon D. Laman, Jack van Horssen, Jan Bauer, Yolanda S. Kap

**Affiliations:** ^1^Department of Immunobiology, Biomedical Primate Research Center, Rijswijk, Netherlands; ^2^Department of Neuroscience, University of Groningen, University Medical Center, Groningen, Netherlands; ^3^Department of Parasitology, Biomedical Primate Research Center, Rijswijk, Netherlands; ^4^MS Center Noord-Nederland, Groningen, Netherlands; ^5^Department of Molecular Cell Biology and Immunology, VU University Medical Center, Amsterdam, Netherlands; ^6^Department of Neuroimmunology, Brain Research Institute, Medical University Vienna, Vienna, Austria

**Keywords:** multiple sclerosis, animal model, demyelination, experimental autoimmune encephalomyelitis, Epstein–Barr virus, B cell

## Abstract

The absence of pathological hallmarks of progressive multiple sclerosis (MS) in commonly used rodent models of experimental autoimmune encephalomyelitis (EAE) hinders the development of adequate treatments for progressive disease. Work reviewed here shows that such hallmarks are present in the EAE model in marmoset monkeys (*Callithrix jacchus*). The minimal requirement for induction of progressive MS pathology is immunization with a synthetic peptide representing residues 34–56 from human myelin oligodendrocyte glycoprotein (MOG) formulated with a mineral oil [incomplete Freund’s adjuvant (IFA)]. Pathological aspects include demyelination of cortical gray matter with microglia activation, oxidative stress, and redistribution of iron. When the peptide is formulated in complete Freund’s adjuvant, which contains mycobacteria that relay strong activation signals to myeloid cells, oxidative damage pathways are strongly boosted leading to more intensive pathology. The proven absence of immune potentiating danger signals in the MOG34–56/IFA formulation implies that a narrow population of antigen-experienced T cells present in the monkey’s immune repertoire is activated. This novel pathway involves the interplay of lymphocryptovirus-infected B cells with MHC class Ib/Caja-E restricted CD8+ CD56+ cytotoxic T lymphocytes.

## Introduction

Multiple sclerosis (MS) is a devastating autoimmune neurological disease that damages the human central nervous system (CNS) through inflammation and tissue injury (1, 2). With an incidence of 1 per 1,000 affected individuals and two million patients worldwide, MS is the most common non-traumatic neurological disorder in young adults (3). The pathological hallmark of the disease and the most likely cause of the neurological deficits is the lesion, a usually well-defined area where axon-enwrapping myelin sheaths are destroyed, a process indicated as demyelination. Lesions typically display a variable degree of inflammation, i.e., infiltration of lymphocytes and monocytes into meninges and CNS parenchyma together with local activation of microglia cells, axonal injury, and proliferation of astrocytes (gliosis) (4). Depending on the disease stage, lesions can be located in the white matter (WM) and gray matter (GM) of brain and spinal cord (5, 6).

The disease course observed in the majority of MS patients (±70%) can be divided into three phases (7): 1. A pre-symptomatic phase without detectable clinical symptoms where intra-CNS focal inflammation can be detected on magnetic resonance images. 2. A relapsing–remitting (RR) phase where episodes of disease activity (relapses) alternate with intermittent recovery (remission). A subset of patients displays clinically isolated syndrome, a usually short-lasting episode with a single neurological deficit (e.g., optic neuritis), as first clinical event. 3. A secondary progressive (SP) phase where clinical symptoms worsen progressively, while remissions become less frequent and ultimately disappear. In a minority of patients (±15%), the disease course is progressive from the onset; this is primary progressive (PP) MS (8). The transition of RR to SPMS cannot be accurately determined as there are no clear clinical, imaging, or immunological parameters that define the transition point (7). One established factor determining the onset of progressive disease is the age of the patient rather than the duration of the antecedent RR disease (9). It is also unclear whether SP and PPMS are identical or different clinical entities (10).

Accumulating evidence indicates profound differences in the underlying pathogenic mechanisms between RRMS and SP/PPMS. The pathogenic process in RRMS is dominated by immune-driven inflammation and demyelination, which can be reasonably well treated with drugs that modulate or suppress immune functions (11). However, such treatments are usually ineffective in progressive MS, indicating that the transition from RRMS to SPMS is associated with a change of the pathogenic mechanism. Our limited understanding of the pathogenic mechanisms in progressive MS and the lack of (a) relevant animal model(s) contribute to the high unmet need of effective treatments for progressive disease. Gaining insight into the rate-limiting steps in progressive MS as a basis of future innovative treatments is, therefore, regarded as the greatest challenge for the MS research community (12).

## Modeling Progressive MS in Animals

Progressive MS is clinically characterized as gradual worsening of neurological functions, which in SP disease manifests after a relapsing disease course. The pathology of RR and progressive MS shows many differences, which are briefly summarized here. For a detailed description, we refer the reader to recent reviews (6, 10).

A prominent macroscopic pathological feature of progressive disease is the loss of brain volume (atrophy), which is mainly due to the degeneration of chronically demyelinated axons. Another pathological feature of progressive MS is demyelination of cortical GM. Intriguing data from Lucchinetti et al. (13) provides compelling evidence that cortical GM lesions are not confined to progressive MS, but can be found also in the early phase of the disease. For the intra- and leukocortical lesions, an important difference between early versus late disease is the presence of inflammation in early MS lesions, while inflammation activity has been cleared in progressive MS. In subpial lesions, located at the cortical surface, inflammation is usually restricted to activation of microglia, although inflammatory cell infiltrates can be found in the adjacent meninges. The activated microglia contribute to tissue injury by the production of various reactive oxygen species and proteases. The chronic oxidative stress in lesions disturbs the redox state in neurons and axons, which leads to mitochondrial dysfunction and ultimately neurodegeneration.

The dominant animal model in the preclinical research of MS is the mouse experimental autoimmune encephalomyelitis (EAE) model. As, by far, the greatest majority of fundamental discoveries in neuroimmunology were done in mice, there clearly is a relevance of mouse models for our understanding of autoimmune mechanisms in MS. There is a plethora of excellent reviews on rodent EAE models in which clinical and pathological aspects as well as their relevance for MS are discussed in much greater depth than can be discussed here (14–18). We like to refer readers with a specific interest in the mouse models of MS to these reviews. Usually, primate EAE models are not discussed in these reviews, although these models can provide useful information about the pathogenesis of MS, which cannot be obtained in rodent EAE models (19). The primary aim of this publication is, therefore, to fill this knowledge gap, with a focus on progressive MS.

Several mouse EAE models have been proposed as being relevant for progressive MS. For example, in the myelin oligodendrocyte glycoprotein (MOG)-induced EAE model in Biozzi ABH mice the disease starts with alternation of relapses with complete remission; but after a certain period of time, residual functional recovery becomes incomplete. MOG-induced models in B6 mice or in non-obese diabetic mice have also been proposed as model of progressive MS. In the mouse models, loss of functional recovery is found associated with failure of immunoregulatory mechanisms, including regulatory T cells, and incomplete repair of demyelination. However, pathological hallmarks of progressive MS, in particular demyelination of cortical GM, oxidative injury, redistribution of iron, and mitochondrial defects, are less common in rodent EAE models than in the human disease or are even absent (20). There are two rat models that display cerebral GM demyelination and are potentially useful for the study of cortical GM demyelination. These are MOG-induced EAE models in Lew.1AR1 and Lew.1W strains (21) and stereotactic injection of inflammatory cytokines (TNF-α/IFNγ) into the cortex of MOG-induced EAE-affected Lew rats (22).

We will discuss here that the mentioned pathological features are present in the EAE model in common marmosets, a small-bodied New World primate species (*Callithrix jacchus*), and that, in this respect, this model more closely approximates the situation in progressive MS than mouse EAE models.

The common marmoset offers a variety of translationally relevant models of human disease (23). Particularly relevant for diseases caused by (auto)immune-mediated inflammatory pathology (AIMID), such as the here discussed neuro-inflammatory disease MS, is the evolutionary proximity to humans, which has been estimated at ±40 million years (24). This close evolutionary distance is reflected by a high degree of genetic and immunological similarity (25). This, added to the outbred nature of the species and the presence of a pathogen-educated immune system, creates a unique experimental animal model for translational research into the pathogenesis and treatment of MS.

Depending on the mode of immunization, a given marmoset EAE model will display key signs of RR or progressive MS pathology. We will discuss neuropathological details of the models as well as the underlying immunopathogenic mechanisms. Intriguingly, a core pathogenic process leading to progressive MS pathology in marmosets is formed by an unconventional pathogenic mechanism that seems to be absent in mouse EAE models. This mechanism involves the cross talk of B cells infected with Callithrichine herpesvirus-3 (CalHV3) and CD8+ CD56+ effector memory cytotoxic T cells, which are reactive with a cytomegalovirus (CMV) antigen (26). Of note, CalHV3 is the marmoset representative within the lymphocryptovirus (LCV) genus LCV, which also comprises the human-specific LCV Epstein–Barr virus (EBV, HHV4), the rhesus macaque exponent rhesus lymphocryptovirus and herpesvirus papio from baboons (27).

## Transition from RR to Progressive MS; A Working Concept Derived from Marmoset EAE

According to a published theory MS is a two-stage disease, starting with RRMS that eventually leads to SPMS (28). The current evidence indicates that RRMS is caused by an autoimmune attack directed at the myelin sheaths, while oligodendrocytes (ODC) are (initially) spared and remyelination remains possible. Progressive MS is thought to be rather caused by age-associated degeneration of ODC and neurons, leading to insufficient repair and irreversible damage to the nervous system. However, data from pathology studies and from recent therapy trials in PPMS, such as the ORATORIO trial testing ocrelizumab, a monoclonal antibody (mAb) against human CD20, a broadly expressed surface marker of B lineage cells, support a role for autoimmune mechanisms also in progressive MS (29, 30).

**Figure 1:**
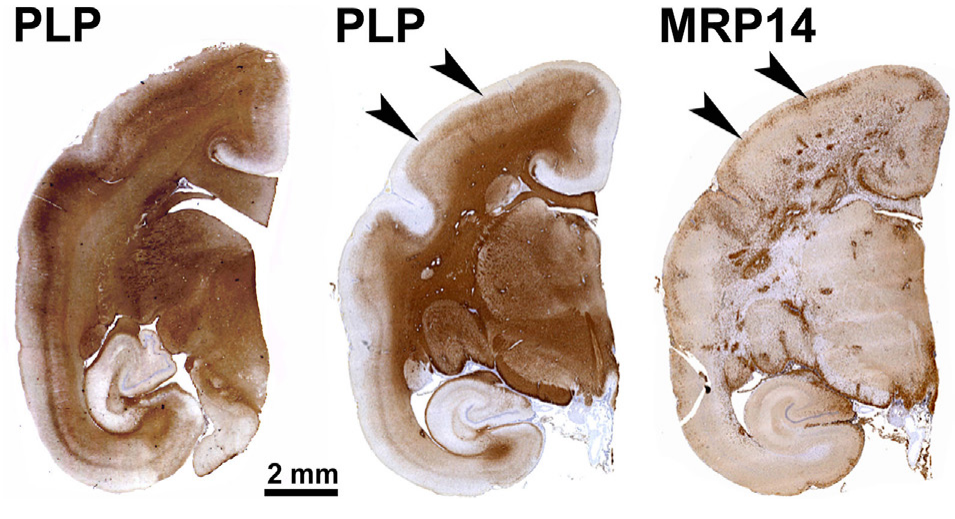
Patterns of demyelination (PLP) and inflammation (MRP14) in a marmoset immunized with multiple sclerosis myelin/complete Freund’s adjuvant. This archetypical experimental autoimmune encephalomyelitis model is characterized by abundant MRP14+ resident (microglia) and infiltrated (macrophages) myeloid cells, which are found in multiple dense clusters in the white matter and as a band at the rim of subpial demyelinated lesions (arrowheads).

A central message of this review is the observation that disease development in the marmoset EAE model follows a similar two-stage course (19). Data obtained over the past two decades provide insight into the disease mechanisms underlying the two disease stages. The first documented induction of MS-like disease in marmosets was by Massacesi et al. (31). In the original protocol, EAE was induced with human myelin, which was formulated with complete Freund’s adjuvant (CFA), followed by intravenous injection of heat-killed *Bordetella pertussis* particles. CFA consists of a mineral oil, indicated as incomplete Freund’s adjuvant (IFA), and heat-killed mycobacteria (*M. tuberculosis* or *M. butyricum*) for potentiation of innate immune mechanisms (32). In our hands, this protocol induced acute and seriously destructive EAE (32). Hence, we modified the protocol to immunization with human myelin, which we isolated from the brain WM of an MS patient, formulated with CFA (32). A single immunization with this formulation elicited a chronic neurological disease that displayed MS-like pathology in the WM and the GM of brain and spinal cord (Figure 1). In subsequent experiments, which are briefly summarized here but discussed in greater detail in the next paragraphs, this model was further refined to elucidate the core pathogenic mechanism(s).

A critical finding has been that autoimmunity against a specific albeit quantitatively minor constituent of MOG is dispensable for the initiation of EAE with CNS myelin but essential for the development of chronic disease (33). To study the key pathogenic role of MOG in chronic EAE in further detail, we immunized marmosets with a non-glycosylated recombinant protein expressed in *E*. *coli*, representing residues 1–125 of human MOG (rhMOG), which was formulated with CFA [see next paragraph; reviewed in Ref. (34)].

**Figure 2:**
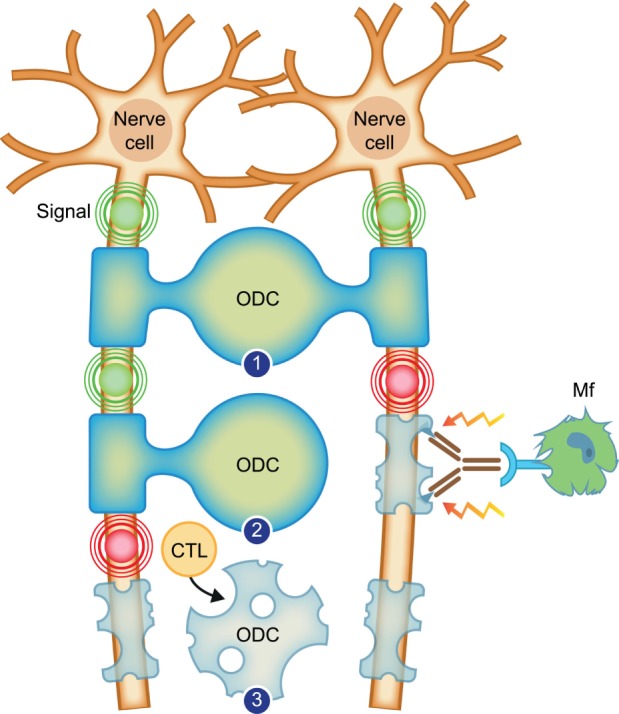
Working concept for the transition of relapsing–remitting multiple sclerosis (RRMS) to secondary progressive multiple sclerosis. Depicted are two neurons, which send electrical signals (indicated with concentric circles) along axons to organs on which they project. The axons are enwrapped with protective myelin sheaths, which are produced and repaired by oligodendrocytes (ODC). (1) Healthy myelinated axons. (2) In *RRMS*, the myelin sheath, in this case of the right axon, is attacked by binding of an antibody, which recruits inflammatory cells, such as the depicted macrophage (Mf). The Mf releases myelinotoxic factors that cause demyelination. New myelin formation (remyelination) is possible as the oligodendrocyte is spared. (3) In *progressive multiple sclerosis*, the myelin-forming oligodendrocyte is killed by cytotoxic T lymphocytes (CTL). Conceptually, this leads to permanent loss of myelination.

The two-stage pathogenic process observed in the rhMOG/CFA model (35) is graphically presented in Figure 2. The collective data (reviewed in subsequent paragraphs) indicate that the initiation phase of the model involves a combined autoimmune attack of pro-inflammatory CD4+ T cells, antibodies, macrophages (Mfs), and complement factors on CNS WM myelin (36). *Via* this pathway, which recapitulates the immunology of mouse EAE models, MS-like lesions are induced in the WM. As remyelination frequently occurred, we assume that the myelin-forming ODC are spared.

After a variable period of time, a second immunopathogenic mechanism is activated, which is mediated by CD8+ cytotoxic T lymphocytes (CTL) (35). In a model based on selective activation of the CTL, we found demyelination in the white as well as the GM of brain and spinal cord in the absence of myelin-binding antibodies, suggesting that demyelination may have been caused by a cytotoxic process that leads to the death of ODC (33).

**Figure 3:**
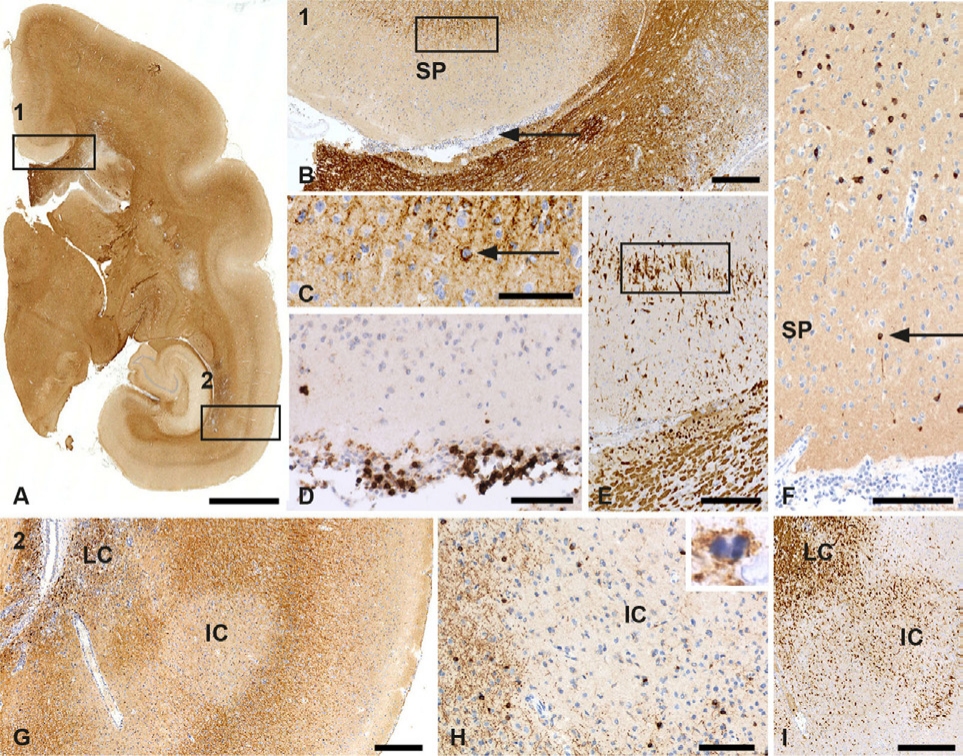
Patterns of gray matter (GM) demyelination in a monkey sensitized against MOG34–56 in incomplete Freund’s adjuvant (IFA). **(A)** Patterns of demyelination (PLP) staining of the right hemisphere of a marmoset with IFA myelin oligodendrocyte glycoprotein experimental autoimmune encephalomyelitis. Rectangle 1 encloses a subpial lesion [enlargement in **(B)**] while the area in rectangle 2 indicates a leukocortical (LC) and intracortical (IC) lesion; an enlargement is shown in **(G)**. **(B)** PLP staining shows the cortex above the corpus callosum. The area indicated by secondary progressive (SP) is a demyelinated GM subpial area. The rectangle here shows the border of the subpial lesion that is enlarged in **(C)**. The arrowhead in this figure points at a meningeal infiltrate. **(C)** In this enlargement, the arrowhead points at a macrophage (Mf) with PLP degeneration products. **(D)** The meningeal infiltrate adjacent to the subpial lesion **(B)** contains a large number of CD3+ T cells. **(E)** Staining for MRP14 shows that cells at the border of the subpial lesion have a microglia morphology. **(F)** Staining for TPPP shows the absence of oligodendrocytes (ODC) in the subpial lesion. The arrowhead points at a single surviving oligodendrocyte. **(G)** PLP staining shows the LC and IC lesion from the areas indexed by the rectangle in **(A)**. **(H)** Staining for CNPase shows the absence of ODC in the center of the IC lesion. The inset shows a PLP+ apoptotic oligodendrocyte at the border of the lesion. **(I)** Staining for MRP14 shows the presence of Mfs/microglia in the LC and IC lesion. Size bars: **(A)** 2 mm; **(B)** 250 µm; **(C–F)** 100 µm; **(G)** 250 µm; **(H,I)** 100 µm.

It was noticed that marmosets immunized with a synthetic peptide derived from rhMOG, representing residues 34–56, henceforth indicated as MOG34–56, formulated with the mineral oil IFA, elicited clinical EAE in >90% of the monkeys (33). The absence of innate stimulatory factors in the peptide–IFA formulation indicates that this highly refined model is based on the reactivation of antigen-experienced T and or B cells present in the healthy marmoset’s immune repertoire. We noticed that in the model induced with MOG34–56/IFA, GM lesions were found, which are devoid of ODC (Figure 3). However, these were usually limited in number and detectable only in a minority (±20%) of the monkeys. We assume that the cases that display GM lesions represent high responders to the immunization.

Gray matter pathology was substantially more robust and present in a higher number of monkeys in the model induced with MOG34–56/CFA, indicating that GM pathology in low responder marmosets can be amplified by innate immune stimulation of myeloid cells by the mycobacteria present in CFA. Lesions in this model are characterized by the activation of oxidative damage pathways, including the redistribution of iron (37). Interestingly, a recent study showed a critical role of CNS infiltrating monocytes expressing C-C chemokine receptor 2 in the formation of cortical GM demyelination in a marmoset EAE model induced with rhMOG/CFA (38). It is tempting to speculate that the stimulation of these myeloid effector cells with mycobacterial antigens in CFA may have amplified GM demyelination in the MOG34–56/CFA model. A direct clinical correlate of CFA has, thus, far not been identified in MS patients. However, previous work shows that a ligand of toll-like receptors (TLR) putatively originating from gut microbiota, i.e., peptidoglycan (PGN) from *Eubacteria*, is imported into the CNS by infiltrating myeloid cells (39). It was shown that Staphylococcus PGN can potentiate the immune response of B6 mice against a non-encephalitogenic challenge with MOG35–55/IFA leading to severe clinical EAE (40).

In conclusion, our findings in the marmoset EAE model support a two-stage model for the immunological events at the transition of RR to SPMS. In the initiation phase of the disease, WM lesions representing those found in RRMS are formed by the combined attack of inflammatory T helper 1 (Th1) cells and anti-MOG antibodies. In the course of the disease, a second pathway is activated mediated by CTL that attack ODC, inducing lesions in WM and GM, which resemble those found in progressive MS. The extent of demyelination is substantially amplified by the concomitant activation of myeloid cells.

## Unraveling the Two Autoimmune Pathways

**Figure 4:**
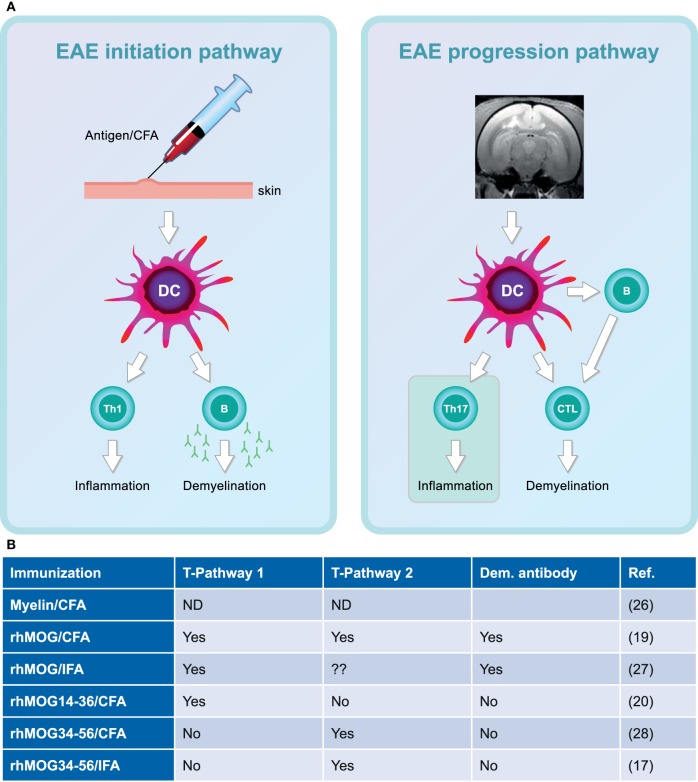
Two-stage immunopathogenesis of experimental autoimmune encephalomyelitis (EAE) in the rhMOG/complete Freund’s adjuvant (CFA)-induced marmoset model. **(A)** The initiation and progression of EAE in rhMOG/CFA sensitized marmosets are driven by distinct T cell subsets. EAE initiation is mediated by the activation of MHC class II/Caja-DRB*W1201-restricted T helper 1 (Th1)/Th17 cells specific for the epitope 24–36 (EAE pathway 1). EAE progression is driven by MHC class I/Caja-E restricted activation of CD8+ CD56+ cytotoxic T lymphocytes-specific for MOG40–48 (EAE pathway 2). **(B)** Overview of the involvement of the two pathways in the different EAE models.

Figure 4 depicts the different pathogenic involvement of T and B cells in the two stages of the pathogenic process in the rhMOG/CFA-induced marmoset EAE model.

### T Cell Involvement

The inoculation of rhMOG/CFA in marmosets initially elicits the activation of Caja-DRB*W1201-restricted CD4+ Th1 cells specific for the epitope MOG24–36. Of note, Caja is the acronym used for the MHC system of marmosets (from *C. jacchus*). The Th1 cells were found to elicit only mild inflammatory lesions in the WM (36, 41), but in conjunction with autoantibodies, binding to a conformational epitope located at the apical side of the molecule, large demyelinated lesions resembling those in RRMS were formed (42). The crucial pathogenic role of the Th1 cells was confirmed by the robust clinical effect of ustekinumab, a human IgG1κ mAb directed against the shared p40 subunit of human interleukin (IL)-12 and IL-23 (43). These factors are produced by myeloid antigen-presenting cells (APC) upon stimulation *via* TLR (44) and skew the differentiation of Th0 precursor cells toward pro-inflammatory Th1 and Th17 functional profiles.

The second autoimmune pathway, for which thus far no equivalent process has been found in mouse EAE models, is activated after a variable period of time following EAE initiation and seems to dictate the EAE progression rate (35). This progression pathway 2 involves the activation of Caja-E restricted CD8+ CD56+ CTL specific for the epitope MOG40–48 which have the capacity to kill target cells pulsed with the MOG40–48 epitope (45). Of note, a similar type of T cells has been found in MS lesions in close proximity of HLA-E+ ODC, indicating a cytotoxic process (46). It is tempting to speculate that the absence of ODC in GM lesions formed in the MOG34–56/IFA model (Figure 3) is due to a similar process, but this needs to be formally proven. Treatment with ustekinumab at a late disease stage only delayed the onset of clinically evident EAE, indicating that Th1/Th17 cells have a less prominent pathogenic role than in the initiation phase of the disease (47).

### B Cell Involvement

Using a fully human mAb against human CD20 (HuMab7D8), which is clonally related to the clinically tested mAb ofatumumab (48, 49), we observed a profound effect of B cell depletion on lesion formation in WM as well as GM in the rhMOG/CFA marmoset EAE model (50, 51). Intriguingly, analogous to the disappointing clinical effect in RRMS of atacicept, a chimeric protein combining the “transmembrane activator and calcium-modulator and cytophilin ligand interactor” TACI, a receptor of the B cell cytokines BlyS (B lymphocyte stimulator) and APRIL (a proliferation-inducing ligand), with the Fc tail of human IgG (52), we observed that depletion of both cytokines with specific mAbs exerted only moderate clinical effects in the EAE model (53). The discrepant clinical effect between the two types of treatment was associated with different depletion patterns of CalHV3 from the lymphoid compartment: the virus was effectively depleted in marmosets treated with the anti-CD20 mAb but not in EAE marmosets treated with mAbs against BlyS or APRIL (45). These and other observations [reviewed in Ref. (26)] lead to the novel concept that the crucial pathogenic role of B cells in the marmoset EAE model may be executed by a small subset of virus-infected B cells, which in humans comprises less than 0.005% of all B cells (54). Experiments are in progress to test whether selective depletion of this subset exerts a sufficient beneficial effect on marmoset EAE. We posit here that this mechanism may also explain the established, albeit still elusive association between EBV infection and MS risk (55).

The crucial role of B cells in the EAE progression pathway was further tested in the highly refined MOG34–56/IFA model in which the autoaggressive CTL are directly activated *in vivo* (33). Also in this model, B cell depletion with the ofatumumab-related anti-CD20 mAb HuMab7D8 exerted a robust effect on the clinical and pathological presentation of EAE, indicating that B cells have a crucial role in the activation of the T cells that cause progressive MS-like pathology and disease (56). As will be explained in following paragraphs, B cells acquire this pathogenic capacity through the infection by LCV.

In conclusion, the EAE model in marmosets involves a two-stage pathogenic process that is initiated by pro-inflammatory Th1 cells and perpetuated by CTL. B cells have a dual role in the disease, namely in the initiation phase the production of autoantibodies that opsonize myelin and activate damage *via* Mfs and complement and in the progression phase presentation of antigen to the CTL. This concept aligns with the recently published remarkable beneficial effect of ocrelizumab, an anti-human CD20 IgG1κ mAb, in progressive MS (30).

## Unraveling the Atypical Marmoset EAE Model Induced with MOG34–56/IFA

An important aspect of primates as model of AIMID is that they are naturally infected with similar viruses and bacteria as those implicated in the shaping of the human immune system, for example, β- and γ-herpesviruses. The important contribution of the environment in the shaping of a human-like immune repertoire in laboratory animals was recently emphasized by the observation that specific pathogen-free (SPF) mice cohoused with pet shop mice, which have a much richer microbial flora than SPF-bred laboratory mice, develop a more human-like immune system (57). Our research in the well-characterized marmoset EAE model suggests that the CTL, which are capable of initiating progressive MS-like pathology in marmoset EAE, may originate from a pathogen-educated part of the immune repertoire (19, 26). This implies that the mechanisms that initiate and/or perpetuate EAE in marmosets differ fundamentally from those driving EAE in SPF-bred mice.

The previously proposed “*response-to-injury*” (inside-out) paradigm for MS (58) was formulated as an alternative for the more widely adhered “*response-to-infection*” (outside-in) concept (59). The essential difference between the two concepts is that in the latter paradigm an infectious micro-organism is the direct trigger of the disease, while in the former paradigm infectious agents make the immune system more responsive to self-antigens released from idiopathic injury inside the CNS WM, indicated as the primary lesion. Primary lesions seem to occur spontaneously without a clear endogenous or exogenous trigger. The exact nature of such primary lesions is unknown, but they could well be the microglia aggregates that are found in the normal appearing WM of MS patients. These aggregates appear in the literature under different names—such as pre-active lesions (60), microglia nodules (60), or newly forming lesions (61)—but may actually represent the same pathological entity (62). It is also well possible that primary lesions are not caused by a pathological event but may rather be due to aging-associated degeneration of myelin (63) or to dietary factors associated with a Western life-style (64).

**Figure 5:**
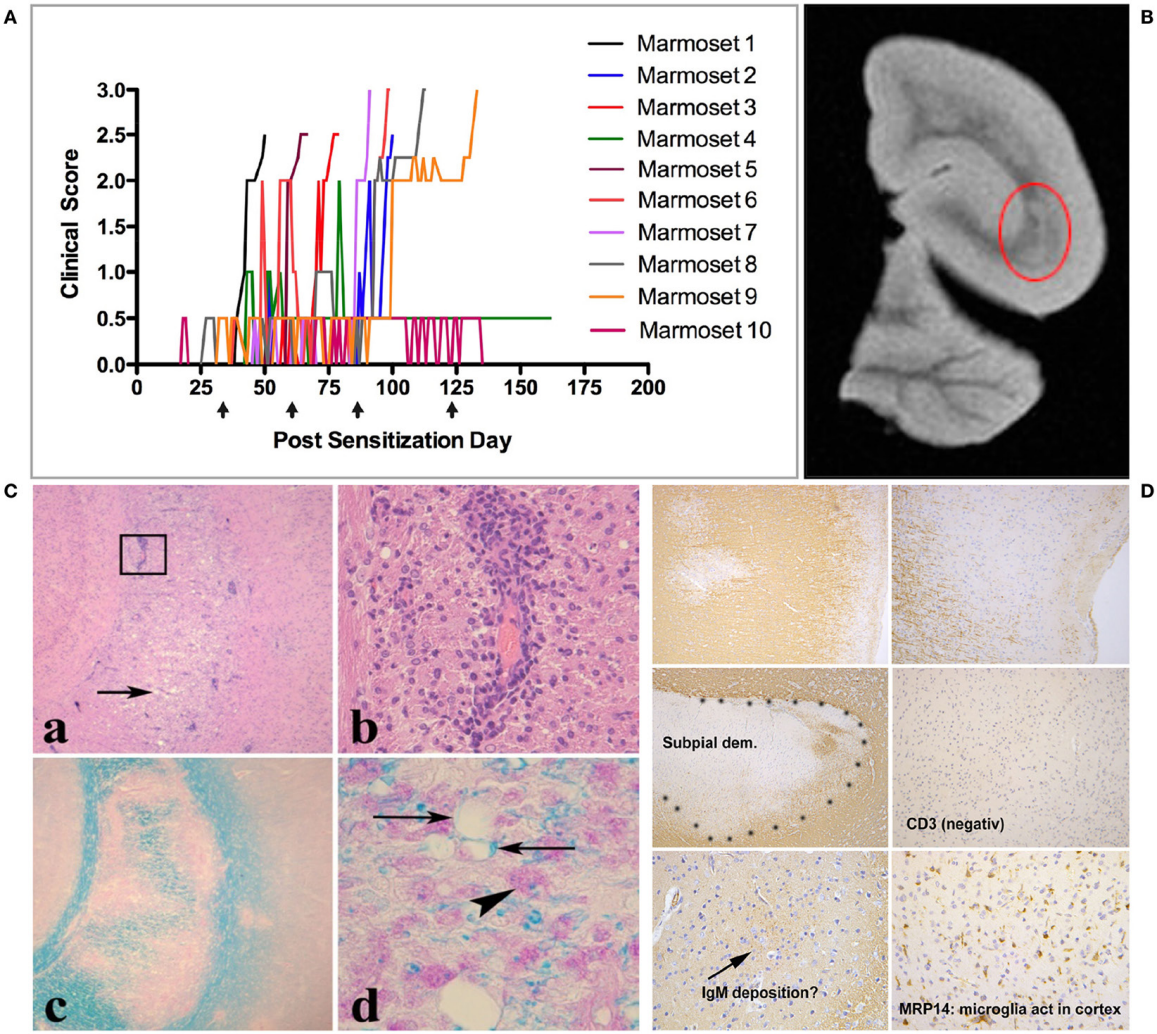
Clinical and pathological aspects of an atypical marmoset experimental autoimmune encephalomyelitis model induced with MOG34–56/incomplete Freund’s adjuvant (IFA). **(A)** 10 unrelated marmosets were immunized at 28 days interval (arrows) with MOG34–56/IFA. Depicted is the heterogeneous disease development, comprising early and late responders. **(B)** A representative T2W magnetic resonance images scan of a formalin-fixed brain half, showing multiple hyperintense regions in the white matter. The encircled lesion (red) was examined for presence of inflammation [**(C)**: a,b] and demyelination [**(C)**: c,d]. **(D)** Demyelinated lesions in the cortical gray matter. In these lesions, mononuclear cell infiltrates and antibody deposition are absent, while prominent activation of microglia is present.

Conceptually, antigens released from these primary lesions are captured by APC within the cervical and lumbar lymph nodes, which, respectively, drain the brain and spinal cord (58). The assumption that EAE progression mechanisms are activated in CNS draining lymph nodes is supported by observations in the MOG EAE model in Biozzi ABH mice (65) but needs to be proven for marmoset EAE. Conceptually, the pattern of immune responses to injury is determined by the composition and activation state of the immune repertoire, which is shaped by the interaction of genetic and environmental factors (58). Accordingly, the pathogen-educated immune system of healthy marmosets comprises autoaggressive CD8+ CD56+ cytotoxic T cells, which can be activated *in vivo* by repeated injection (between 1 and 4 at 28 days interval) of human MOG-derived peptide 34–56 with the mineral oil IFA as adjuvant (33). Immune profiling showed absence of myelin-binding antibodies in this model and a narrow cellular response, comprising CD4+ CD56+ and CD8+ CD56+ T cells specific for the immunizing MOG34–56 peptide (33, 66). Figure 5 shows the variable clinical response (Figure 5A) and the lesion load in the cerebral WM (Figure 5B). At closer histological examination, inflammation and demyelination can be observed in the cerebral WM (Figure 5C) and, albeit only in a subset of the monkeys (±20%), in the GM (Figure 5D). The observation that the same formulation was completely inactive in MOG EAE susceptible SPF-bred mice, such as Biozzi ABH and C57BL/6 (33), indicates that the EAE in marmosets had been induced by antigen-experienced T cells, which are apparently present in the healthy marmoset’s immune system.

A recent study shows that the immune response elicited by the immunization with MOG34–56/IFA is qualitatively heterogeneous, possibly reflecting genetic differences outside the MHC region between the monkeys (67). We observed only in high responder monkeys, which were characterized by a fast EAE progression rate, a clinical effect of IL-7 receptor blockade with an anti-human CD127 mAb. This is a potentially important finding as polymorphisms in the IL-7 receptor promotor region are associated with enhanced MS risk (68). Moreover, Bielekova et al. reported that IL-7 responsive (CD4+) T cells specific for MOG35–55 are enriched in the blood of MS patients compared to healthy controls (69).

## B-T Cross Talk in the MOG34–56/IFA Model

The variety of GM lesion types that present in the marmoset EAE model (subpial, intracortical, transcortical) (Figure 3) mirrors those found in the MS brain. A recently published analysis shows that lesions in the WM as well as those in the GM display clear signs of oxidative injury, including activation of the NADPH oxidase, redistribution of iron, and oxidant damage to lipids and DNA (37). The absence of these features in mouse EAE models implies an important translational gap with MS (20). This difference is rather remarkable as it occurs irrespective of the large difference in innate immune stimulation between the mouse EAE models, involving strong immune potentiation with the adjuvants CFA and *B. pertussis*, and the marmoset model EAE, which is induced with a TLR-ligand-free emulsion of a synthetic peptide in IFA.

We posit here that the marmoset EAE model fills the gap between mouse EAE models and progressive MS. The model can, therefore, be used for developing effective treatments for aspects of the disease that cannot be treated with current medications, such as the primary and SP forms of MS. Intriguingly, the model supports a core pathogenic role of LCV-infected B cells in progressive MS. The question arises why LCV infection is crucial for this pathogenic role of B cells.

It is highly intriguing that a sequence of only 23 amino acids (GMEVGW**YRPPFSRVV**HLYRNGKD; underlined is the core epitope MOG40–48) formulated with an adjuvant without innate stimulatory activity gives sufficient instruction to the marmoset immune system for eliciting the pathologically complex MS-like autoimmune disease depicted in Figure 3. The observation that this formulation is inactive in SPF-bred mice (33) suggests that the observed effects in marmosets may be due to their pathogen-educated immune system.

A recent study shows that two types of messages are relayed from LCV-induced B lymphoblastoid cells (BLC) to T cells, namely those involving the tri-molecular complex of MHC class I or II molecule—epitope—T cell receptor and those exchanged without cognate epitope recognition (70). Signals relayed from the BLC that circumvent antigen recognition include linkage of CD70, which is strongly upregulated on BLC, with CD27 on T cells, causing downregulation of the latter on CD4+ and CD8+ T cells. Moreover, we observed reduced expression on T cells of CD127, the receptor of IL-7 on T cells, associated with increased expression of CD95 and PD1 (CD279). In this context, it is interesting to note that blocking of the IL-7R with an anti-human CD127 mAb abrogated fast EAE development in the MOG34–56/IFA model (67). The above-described cellular changes induced in LCV-infected B cells may need to be viewed as an immune escape strategy of the virus (71, 72). Interestingly, the production of IL-17A, which is the signature cytokine of the MOG34–56/IFA induced marmoset EAE model (33), completely depended on the presence of MOG34–56 in the culture.

The presence of strongly autoaggressive T cells in the immune repertoire of healthy monkeys indicates that these either have escaped thymic selection or have been induced by antecedent exposure of the marmosets to mimicry epitopes expressed by an infectious agent. As will be discussed below, these options are not necessarily mutually exclusive. In the model induced with MOG34–56/IFA, we observed a dominant role of CD8+ CD56+ effector memory CTL, which are restricted by the non-classical *Mhc class I^b^* allele *Caja-E* and specific for the epitope MOG40–48 (YRPPFSRVV) (45). Analysis of serum antibodies revealed that specificities binding myelin particles were absent (66), indicating that the autoaggressive CTL may be held directly responsible for the observed demyelination of WM and cortical GM in this model. The MOG40–48 epitope shares sequence identity as well as immunological cross-reaction with the major capsid protein of CMV, an immunodominant T cell antigen in the human population encoded in the UL86 open reading frame (73, 74). The restriction of antigen recognition by Caja-E, the expression of a natural killer (NK) cell marker (CD56), and the cross-reaction with a peptide derived from an immunodominant CMV antigen are reminiscent to NK-CTL involved in the control of CMV infection in humans (75). As was already mentioned, a similar type of T cells, albeit of different specificity, has been identified in MS lesions, where they seemed to be engaged in a cytotoxic interaction with HLA-E expressing ODC (46). Taken together, these observations led us to posit that pathology in this model may be caused by autoaggressive T cells related to human NK-CTL which cause demyelination by cytotoxic killing of ODC.

Regarding the recognition of age as most important risk factor in progressive MS (9), it is noteworthy that chronic latent CMV infection has been held responsible for age-associated changes in the human immune system, such as the expansion of CD8+ CD28null T cells, which have been implicated in chronic autoimmune inflammation and progressive MS development (76–79). Note also that a relevant proportion of the surprisingly high frequency of human anti-CMV effector memory T cells in the aging human immune repertoire is directed against the UL86 antigen of CMV (74).

## A Critical Role of Memory B Cells in MS

For many years, the B lymphocyte has been assigned a subdominant role in the pathogenesis of MS, namely as producers of autoantibodies that opsonize myelin and mediate demyelination *via* cytotoxic mechanisms involving complement (CDC) or Mfs (ADCC). The humble position of the B cell is rather remarkable as EBV, a virus that primarily targets B cells, had already been mentioned as a possible trigger of MS in an editorial in the Lancet of 1976 (80). The observation that depletion of B cells with rituximab, a chimeric mAb directed against human CD20, had a profound and persistent suppressive effect in RRMS without essentially altering circulating antibody levels caused a paradigm shift (81). The research following this enigmatic finding shows that B cells have many more key functions in MS than only autoantibody production; they also produce cytokines, present autoantigen to T cells, are the primary target of EBV, which they transfer into the CNS and (according to some) organize the ectopic lymphoid structures found in the MS brain (82–84). For a detailed description of the pathogenic role of B cells in the MS pathogenesis, we like to refer the interested reader to the many excellent reviews on this subject (16, 49, 85, 86). An important final note is that the pathogenic role of B cells in MS may be restricted to a subset, such as memory B cells (16) or the EBV-infected subset (26). This implies that therapies could be developed that target only the pathogenic subset of B cells, thereby sparing the rest of the repertoire.

## LCV Infection Empowers B Cells for Autoimmunity Induction

While EAE is experimentally induced by one or more injections of antigen/adjuvant emulsion, MS seems to develop spontaneously without a clear environmental trigger. It is, therefore, an important question where and how the autoaggressive NK-CTL discussed in the previous paragraph are activated in the MS patient.

We proposed that MS starts with an idiopathic primary lesion within the CNS, which in individuals prone to develop MS elicits an autoimmune process (58). However, work by Cserr and others showed that antigens released from injured CNS myelin and captured in draining lymph nodes rather elicited Th2-type anti-inflammatory than Th1/Th17-type pro-inflammatory T cell responses (87). On the other hand, in the context of EAE, pathogenic T cell responses against antigens released from CNS myelin injury may be enhanced in CNS draining lymph nodes (65, 88). This paradox can be understood when post-translational modification of myelin antigens in the inflammatory milieu of MS lesions and/or the involvement of APC with particular pathogenic capacities are taken into account. Both possibilities will be further discussed below.

Reverse translation analysis of immunotherapies that were or were not clinically effective in MS clinical trials pointed to a crucial role of CalHV3-infected B cells in the activation of the autoaggressive CTL in this model [for review, see Ref. (26)]. Regarding the strong, albeit still elusive, association of EBV with MS this is a potentially important finding (89). Recent work provided insight into the essential role of the virus in the pathogenic process. It was found that the infection with LCV endows B cells with the capacity to cross-present the proteolysis-sensitive MOG40–48 epitope from the encephalitogenic MOG34–56 peptide to MHC-E restricted CTL (90). How might this work?

According to an immunological dogma, autoreactive T cells are eliminated from the immune repertoire by negative selection in the thymus (=central tolerance) against tissue restricted antigens expressed in thymic epithelial cells under the control of the autoimmune regulator (91) or self-antigens imported by thymus infiltrating dendritic cells and B cells (92, 93). Nevertheless, presence of potentially hazardous autoaggressive T cells in the healthy repertoire has been firmly established (94). A plausible albeit poorly examined explanation for the escape of certain autoaggressive T cell specificities from negative selection in the thymic medulla may be that these are specific for protease-sensitive epitopes that are cleaved during processing into MHC binding peptides (epitopes) by thymic APC; this has been demonstrated for myelin basic protein (MBP) (95).

We hypothesized that in healthy individuals, activation of these escaped T cells in the periphery might be prevented by destructive processing of these same epitopes in peripheral APC. Mutatis mutandis, this would imply that in MS patients this peripheral tolerance mechanism might be impaired when B cells are infected with EBV. We obtained evidence that this may indeed be the case for the MOG40–48 epitope of the MOG-specific CTL that drive disease progression in the marmoset EAE model discussed here (90). In brief, we found that the virus activates two essential mechanisms in B cells, namely protein citrullination and autophagy, *via* which the peptide is protected against fast degradation (90). Of note, citrullination, the conversion of arginine into citrulline (Citr), is a frequently occurring post-translational modification of autoantigens in the MS lesion (96) that seems to be relevant for chronic EAE development in mice (97).

In their putative role as APC for the autoaggressive CTL, the LCV-infected BLC face the complex challenge to present the MOG40–48 epitope to the autoaggressive CTL, while at the same time they need to suppress expression of viral antigens to avoid detection by the host immune system. The latter is achieved by prohibiting epitope loading on MHC and surface expression of MHC class Ia molecules (98). The solution for this paradox might be that MHC-E molecules are used for the presentation of self and viral peptides. A similar dichotomous role of HLA-E is exploited by the β-herpesvirus CMV, which just like EBV achieves immune escape by downregulating MHC class Ia molecules (HLA-A, B, C) and simultaneously upregulating HLA-E to avoid detection and kill by NK cells (99). It was found that depending on the peptide present in the cleft, HLA-E binds with inhibitory natural killer group (NKG) 2A/CD94 receptors expressed by NK cells, or permits cytotoxic activity by the binding of a dimer consisting of NKG2C and CD94 (100). In the case of CMV, binding of the leader peptide of gpUL40 antigen directs HLA-E binding with NKG2A/CD94 dimers for immune evasion (101). On the other hand, the human immune repertoire contains HLA-E restricted NK-CTL with which virus antigens, including peptides derived from gpUL40, are detected for immune protection (102). Using a competition assay with a leader peptide of HLA-G, we could demonstrate binding of the MOG40–48 epitope to K562 cells expressing Caja-E or HLA-E transgenes. In addition, we confirmed cytotoxic activity of anti-MOG40–48 marmoset T cells toward peptide-pulsed autologous EBV-infected marmoset B cells (45).

Studies on the processing of the rhMOG in human B cells showed that the leading protease in endolysosomal degradation is cathepsin G (catG) (103), a serine protease involved in antigen processing by B cells (104). Remarkably, B cells display significant catG activity although expression of catG mRNA could not be detected with conventional PCR (105). A similar observation was published by Burster et al. (106). As catG cleaves the immunodominant peptide MOG34–51 at the arginine residues on positions 41 and 46 (Arg^41^ and Arg^46^), the MOG40–48 epitope of the CTL is destroyed during processing. Intriguingly, citrullination of only the Arg^46^ residue sufficed for protection of the complete MOG35–51 peptide against proteolytic degradation, although the peptide contains at least five other potential cleavage sites, including the Arg^41^ residue (107). It was shown that modification of the Arg^46^ is crucial for association of the epitope with the autophagy pathway, which provides a virus-induced protection mechanism for the peptide (103). The importance of citrullination of the Arg^46^ residue may lie in the fact that this residue is located within a putative F-LIR motif (FSRV) *via* which the peptide can interact with the LC3 docking molecule of autophagosomes (105).

**Figure 6:**
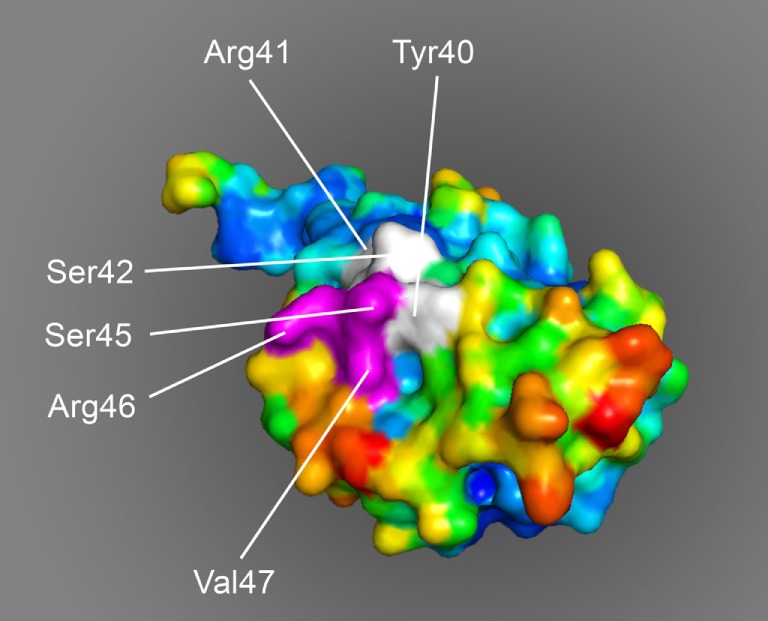
Space-filling model of monomeric myelin oligodendrocyte glycoprotein (PDB accession number 1PKO) in molecular surface representation, colored according to B-factor (blue, low rms/rigid; red, high rms/flexible). The surface-exposed MOG40–48 epitope (YRSPFSRVV) is indicated in white/purple. The P43 and F44 residues, which stick out of the plane toward the reader, are not resolved in the structure, probably due to the high flexibility of this part of the sequence resulting in a diffuse diffraction pattern (109), the V48 residue is buried in the interior of the protein and, therefore, not visible. The putative LIR-motif (F_43_SRV_47_), which is part of the 40–48 epitope is shown in purple. The surface exposure of this motif enables interaction with the LC3 docking molecule of autophagosomes.

The molecular modeling of monomeric MOG shows that the MOG40–48 epitope is surface-exposed and is readily accessible for by the peptidylarginine deïminases that catalyze the conversion of Arg into Citr (Figure 6). The association of the MOG35–51 peptide with LC3 in autophagosomes may not only protect the pathologically most relevant MOG40–48 epitope against proteolytic degradation but may also bring the epitope in close contact with MHC-E molecules, which according to a recent paper also associate with LC3 (108).

## Concluding Remarks

A final and obviously highly relevant point of discussion is to what extent the pathological mechanisms defined in the marmoset EAE models can be extrapolated to the pathogenesis of MS. In a seminal review, Stys et al. posited the intriguing concept that PPMS is the real MS, which is putatively caused by degenerative events (110). This would imply that the induction of RRMS is a secondary pathogenic event. On the other hand, the recently completed ORATORIO trial indicates a pathogenic role of B cells in PPMS (30). Data obtained in the marmoset EAE revealed that the two concepts can be merged.

We propose that in MS the MHC-E restricted NK-CTL that drive EAE progression *via* pathway 2 are triggered early in the disease by myelin antigens released from an idiopathic primary lesion, e.g., a microglia nodule, with a key role of EBV-infected B cells in the presentation of released antigen to the CTL. The fact that the MHC-E loci in humans and non-human primate are essentially invariant may explain why the effect of MHC-E genes on MS risk has not emerged in GWAS studies. In line with our proposal Zaguia et al. reported presence of CTL, albeit specific for another myelin antigen (MBP) in RRMS lesions in close proximity of HLA-E expressing ODC, which seem to undergo a cytotoxic attack (46). Although the lack of cross-reactive mAbs has hampered the full characterization of the autoaggressive IL-17+ve CTL that drive the progression pathway in marmoset EAE, the available evidence suggests that they may be related to or even identical with CD8+ CD161+ CD28− NK-CTL present in the human anti-CMV T cell repertoire; a similar T cell type has been implicated in MS (111). The activation of the progression pathway depends on presentation of the proteolysis-sensitive epitopes MOG40–48 by LCV-infected B cells. As discussed elsewhere (76), these vigilant CTL are characterized by reduced sensitivity to immune regulation by Treg cells and are insensitive to corticosteroids (76). Moreover, being antigen-experienced T cells, they are likely committed to a functional lineage and, therefore, refractory to treatments operating at the level of T cell activation and differentiation. This notion may explain the failure of immunomodulatory treatments in progressive MS.

**Figure 7:**
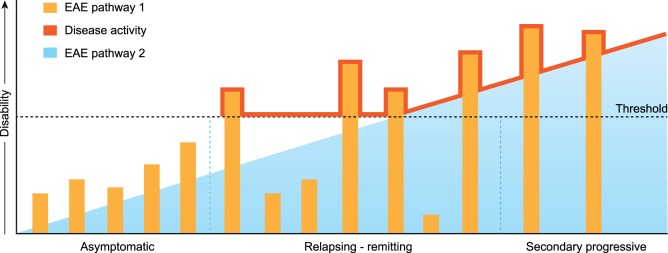
Extrapolation of marmoset experimental autoimmune encephalomyelitis (EAE) pathways 1 and 2 toward multiple sclerosis. The release of myelin antigens from a conceptualized “primary lesion” elicits the activation of distinct EAE mechanisms driven by CD4+ and CD8+ T cells (see Figure 2). The activation of CD8+ cytotoxic T lymphocytes by myelin oligodendrocyte glycoprotein processed and presented by Epstein–Barr virus-infected B cells induces progressive demyelination by the killing of oligodendrocytes (EAE pathway 2). It is proposed that the ensuing excessive release of myelin antigens elicits in genetically prone individuals the activation of pro-inflammatory CD4+ T helper 1 (Th1)/Th17 cells which evoke episodic inflammation-based neurological dysfunction (pathway 1). The ensuing clinical symptoms are indicated with the red line.

As one oligodendrocyte forms multiple (up to 50) myelin sheaths, it can be envisaged that the death of already one oligodendrocyte evokes the release of a substantial amount of myelin antigens. The marmoset EAE model shows that the release of MOG can induce the activation of MHC class II restricted pro-inflammatory CD4+ T cells (112). There is ample evidence in the MS literature that certain alleles of the highly polymorphic MHC-DR locus are strongly linked to enhanced susceptibility for MS. There is also evidence that regulatory mechanisms controlling pro-inflammatory CD4+ T cells are disturbed in MS (113). It can, thus, be envisaged that in genetically prone individuals, episodic activation of Th1/Th17 cells can occur, which elicits exacerbations of neurological dysfunction superimposed on an underlying degenerative process, as depicted in Figure 7. The marmoset EAE pathway 1 complies with such a mechanism for MOG. Importantly, CD4+ T responses in EAE pathway 1 are not necessarily restricted to MOG as shown in Biozzi mice and marmosets sensitized against MOG-deficient myelin (114, 115).

In conclusion, we present here a novel pathogenic mechanism that leads to progressive MS pathology. As reviewed elsewhere this new mechanism may also provide a mechanistic explanation for the elusive association for the association of EBV and MS risk. We posit that in the beginning of the disease the progressive MS pathology may not have direct clinical consequences as it remains below a clinical threshold (Figure 7). However, short-lasting bursts of inflammation triggered by CD4+ T cells reacting against released myelin antigens can cause episodic disturbance of neurological functions (relapses). In this concept, the conversion of RR to SP disease can be viewed as the gradual extinction of pathway 1 activity and incrementing relevance of pathway 2.

## Author Contributions

BH wrote the article and is corresponding author. JD wrote part of the article. BF wrote parts of the article and provided Figure 6. JL had valuable contribution to the underlying concept and performed final editing of the manuscript. JH wrote parts of the article and had valuable contribution to the underlying concept. JB had valuable contribution to the underlying concept; he also provided Figures 1 and 3. YK (senior author) wrote parts of the article and had valuable contribution to the underlying concept.

## Conflict of Interest Statement

The authors declare that the research was conducted in the absence of any commercial or financial relationships that could be construed as a potential conflict of interest.
